# From leaves to microbes: How diet and season shape the fecal microbiome of captive Coquerel´s Sifakas (*Propithecus coquereli*)

**DOI:** 10.3389/frmbi.2025.1680152

**Published:** 2025-11-27

**Authors:** Jonas Schweikhard, Andreas Pauly, Paul Wilhelm Dierkes, Franziska Zoelzer

**Affiliations:** 1 Bioscience Education and Zoo Biology, Goethe University Frankfurt, Frankfurt, Germany; 2 Tierpark Berlin-Friedrichsfelde GmbH, Berlin, Germany

**Keywords:** microbiota, seasonal changes, Oxford Nanopore sequencing, species conservation, nutrition, *Propithecus coquereli*

## Abstract

The community of microorganisms occurring in the animal gut, known as the gut microbiota, is closely connected to host health. It is essential for metabolic processes, digestion, and defense against pathogens. Knowledge about the composition of an intact gut microbiota, along with its natural fluctuations and diversity, is a crucial aspect of proper husbandry and breeding of animals in human care. In this study, we analyzed the fecal microbiota of the critically endangered *Coquerel’s* sifaka (*Propithecus coquereli*), with a special focus on seasonal effects and the impact of dietary variations. As a tropical species, European winters may influence microbiota diversity or composition, highlighting the importance of this assessment. Ninety-seven fecal samples collected from all individuals housed in European zoos revealed high microbial diversity and variation. Some of the core taxa present in every sample included Lachnospiraceae, Erysipelotrichaceae, Clostridiaceae, and Bacillaceae. Microbial α-diversity showed no decline in winter, indicating no seasonal effect caused by dietary changes. However, results suggest compositional differences between seasons, indicated by significant differences in β-diversity. These findings confirm the importance of longitudinal studies to fill knowledge gaps between sampling intervals and to characterize microbiota oscillations throughout the year.

## 1 Introduction

The recent increase in research on the microbiota of various mammalian species (de Jonge et al., 2022; Moeller and Sanders, 2020; Zoelzer et al., 2021) has revealed its importance for host health and wellbeing (Hou et al., 2022; Peixoto et al., 2021) and has shown how an intact gut microbiome may assist the host immune system in avoiding pathogen colonization (Stecher and Hardt, 2011). Interactions between diet and gut microbiota, such as the conversion of dietary fiber into short-chain fatty acids (SCFA) (Koh et al., 2016), provide great insight into the link between food items, the digesting gut microbiota and host metabolism functionality. As the metabolic activity of microorganisms in the mammalian gut is influenced by the host’s diet (Albenberg and Wu, 2014), dietary analysis is an important aspect of microbiome studies.

Microbiome research may also be used as a tool in breeding programs for endangered species in human care. An association between specific microbial taxa and hormone production, as well as breeding success, in the critically endangered eastern black rhino has been shown, revealing the possible use of microbiome analysis in species conservation (Antwis et al., 2019). Recent findings indicate that the sampling season, as well as changes in food resources caused by seasonal variation, might shape the composition and diversity of primate gut microbiota (Baniel et al., 2021). This calls for longitudinal studies that assess not singular fecal samples but rather use time series sampling to analyze both the natural fluctuations over time and the impact of seasonal changes. Incorporating samples from the European winter is of particular interest, as the tropical native range of the species creates a warm climate to which sifakas are adapted. Therefore, the influence of cold climates must be assessed.

The folivorous Coquerel’s sifaka (*Propithecus coquereli*) is a lemur species endemic to Madagascar and considered to be critically endangered (Louis et al., 2020). Given its conservation status, successful breeding in captivity poses a valuable measure to prevent species extinction in human care (Vermeer and Baumeyer, 2022) Therefore, this study aims to characterize the fecal microbiota of all *Propithecus coquereli* individuals housed in European zoos with regard to its diversity, composition, and fluctuation over time. Understanding the baseline composition and natural diversity of the gut microbiome of a species may be useful for detecting disturbances and diseases that can cause severe health issues (McKenney et al., 2017). Therefore, we aimed to analyze not only bacterial but also archaeal and fungal taxa to provide broader coverage of the fecal microbiota. Hence, Oxford Nanopore sequencing was chosen for this study, as it provides longer read lengths and allows for more accurate taxonomic assignment.

While all individuals received an assortment of fresh foliage during summer months, the winter diet was supplemented with defrosted foliage that was harvested and frozen during summer. Due to the known impact of specific dietary items on the lemur gut microbiome (McManus et al., 2021), we analyzed the impact of frozen foliage. The specialized folivores are particularly susceptible to changes in the dietary foliage they consume, with a diverse blend of leaves resulting in higher gut microbial diversity (Greene et al., 2018). The social group that individuals live in affects the composition, making the members of a group more similar to each other at one sampling time than samples of the same individual collected in different years (Perofsky et al., 2021). There are high levels of microbial diversity and an increased abundance of methanogenic archaea in captivity (Greene et al., 2021).

Breeding has so far remained unsuccessful in European captivity, and the husbandry and management of the species have yet to be studied thoroughly. Therefore, to support ongoing conservation efforts for *Propithecus coquereli* in the EAZA (European Association of Zoos and Aquaria), we conducted an assessment of the seasonal, individual, and dietary influences on the species’ fecal microbiota.

## 2 Materials and methods

### 2.1 Sample collection

This study aimed to assess seasonal differences in fecal microbiota diversity and fluctuations in *Propithecus coquereli* based on the dietary changes between summer and winter. In 2022, seven individuals of this species were housed in EAZA member zoos, and fecal samples were acquired from all of these. Sampling was conducted in 2022, once during winter and once during summer. Each time, daily fecal samples were collected for 14 consecutive days, if possible. Sampling was conducted by zoo staff and processed by the authors. Summer samples from one zoo were lost due to transportation difficulties, decreasing the total number of usable fecal samples. A total of 97 fecal samples were obtained. Two zoos housed two individuals each, and one zoo housed three sifakas. Five of these were sampled individually. However, in one zoo, individual sampling was impossible and group samples were obtained. Throughout this study, the total number of fecal samples per individual is referred to as an “individual.” However, the feces of two individuals in one of the study sites could not be assigned to an individual properly, and therefore pooled samples were obtained daily in this case. Sampling was conducted twice a year, with total seasonal samples referred to as a “time series,” both for pooled and individual samples. During these time periods, the animals were fed according to their regular feeding protocol. The regular diet consisted mainly of foliage from locally sourced trees, a variety of vegetables, some nuts, and leaf-eater pellets. The non-invasive sampling was performed by zoo personnel during daily maintenance activities and frozen immediately after collection in sterile 50 mL centrifuge tubes. Each sample was then subsampled into a 1.8 µL cryotube and stored in liquid nitrogen until DNA extraction. The care and use of animals during the research adhered to the research guidelines set by EAZA. After arriving at the laboratory, DNA extraction from each sample was performed using the QIAamp PowerFecal Pro DNA Kit (Qiagen, Hilden, Germany), with subsequent DNA quantification using the Qubit 4 fluorometer (Thermofisher, Massachusetts, USA).

### 2.2 DNA sequencing and data processing

Oxford Nanopore Technologies (ONT) sequencing was performed using the Rapid Barcoding Kit (SQK-RBK004) for library preparation and the Oxford Nanopore MinIon equipped with Oxford Nanopore Flongle Flow Cells R9.4.1 (Oxford Nanopore Technologies, Oxford, United Kingdom). Sequencing was conducted using MinKnow (version 24.02.10), creating FASTQ files. Sequencing adapters were removed from each file using the porechop tool (Wick et al., 2017). The tool NanoFilt (de Coster et al., 2018) was utilized to filter out sequences with fewer than 300 bp as well as quality scores <11. The filtered and trimmed sequences were prepared for taxonomic identification by converting them into FASTA files with the seqtk tool (Li, 2012) and matched with the NCBI databases operated by the tools KRAKEN2 (Wood et al., 2019) and KAIJU (Menzel et al., 2016). Specifically, we used a subset of the NCBI BLAST nr database containing all proteins belonging to archaea, bacteria, and viruses, as well as the fungi, archaea, and protozoa sequences from the NCBI RefSeq database. The taxonomic output was subsequently merged, and features with a count <10 were discarded. The following statistics were performed in R version 4.3.1 (R Core Team, 2023) using the packages vegan (Oksanen et al., 2022) and FSA (Ogle et al., 2023) and phyloseq (McMurdie and Holmes, 2013).

First, the dataset was tested for normal distribution using the Shapiro test (Shapiro and Wilk, 1965), and then taxonomic composition was tested for differences between each time series and season of sampling. Next, alpha diversity was assessed by calculating Shannon index and richness. The calculated diversity indices were then tested for significant differences. Therefore, the Kruskal–Wallis test (Kruskal and Wallis, 1952) and a subsequent Dunn test (Dunn, 1964), Bonferroni corrected was applied to the dataset. Lastly, beta diversity was assessed via permutational multivariate analysis of variance (PERMANOVA). To assess the seasonal impact, we discarded all time series datasets that were from only one season. Only those for which both summer and winter samples were available were included in this assessment.

## 3 Results

### 3.1 Sequence data analyses

**Figure 1:**
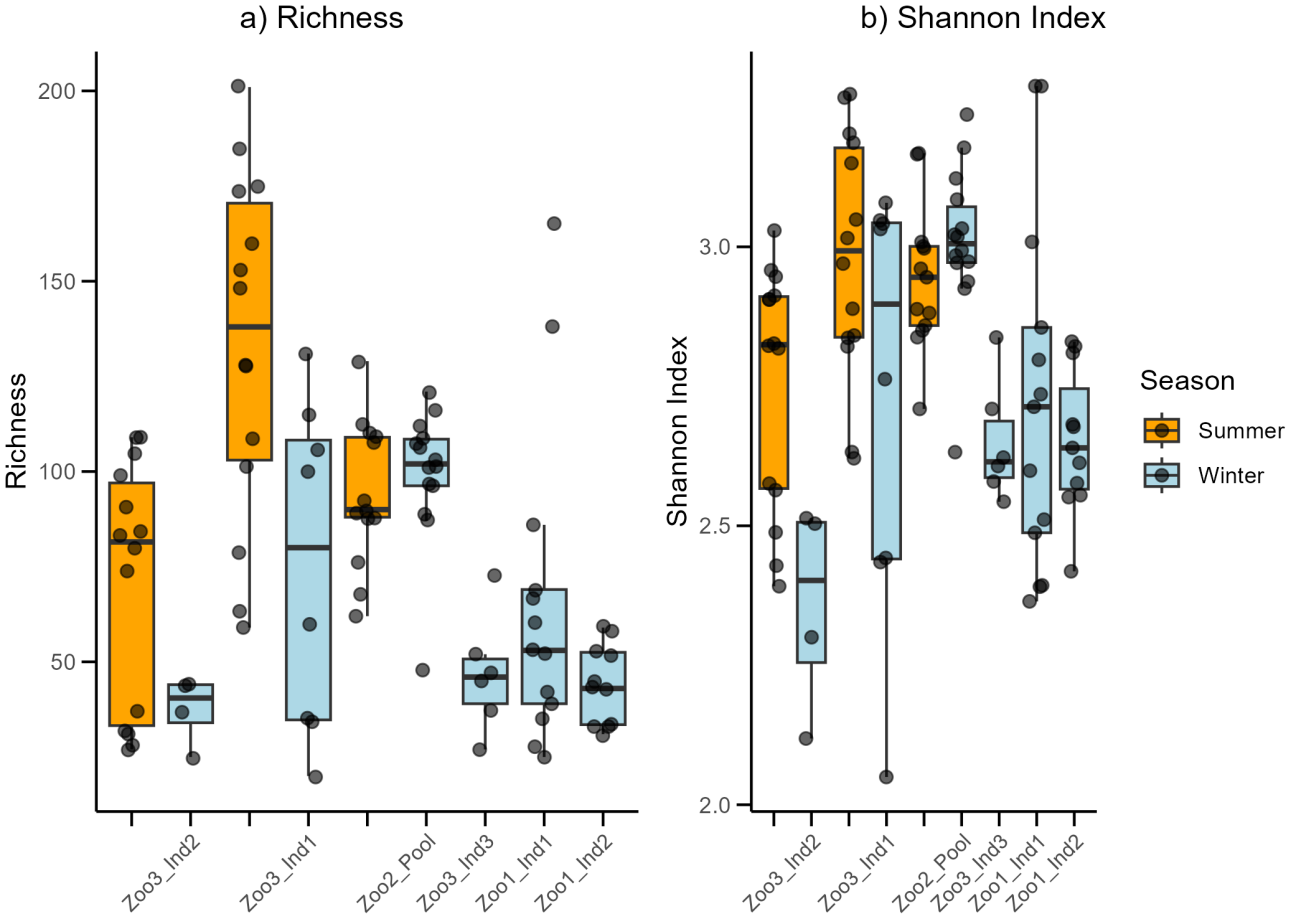
The Richness **(a)** and Shannon index **(b)** for each assessed time series data set. Colors refer to either winter (blue) or summer (orange) samples.

In total, 97 samples from seven *Propithecus coquereli* individuals were analyzed using the Oxford Nanopore sequencing method. After processing the raw data, the dataset consisted of 889,950 sequences, with 2,051–29,533 sequences per sample and an average sequence count of 9,174.74 per sample. The average mean read quality over all samples was 12.52 ± 0.54, and N50 was on average 2,965 ± 965. Overall, a total of 251 ASVs (microbial families) were detected in the dataset, ranging from 20 to 201 features per analyzed sample, with an average of 80.8. In **Figure 1**, the time series are depicted with their respective diversity indices. The Shapiro–Wilk test indicated that the richness values were not normally distributed (*p*<0.01), whereas the Shannon index values of the dataset were normally distributed (*p*=0.061). The Kruskal–Wallis test revealed significant differences in richness between the time series datasets (Kruskal–Wallis: chi-squared=44.86, *p*<0.001). A deeper understanding was achieved using the Dunn test, Bonferroni corrected, which indicated significant differences between Zoo1_Ind1_wi and Zoo3_Ind1_su (p: <0.01), Zoo1_Ind2_wi and Zoo3_Ind1_su (p: <0.01), Zoo3_Ind2_su and Zoo3_Ind1_su (p: 0.023), Zoo3_Ind2_wi and Zoo3_Ind1_su (p: <0.01), Zoo1_Ind2_wi and Zoo2_pool_su (p: 0.025), Zoo1_Ind2_wi and Zoo2_pool_wi (p:<0.01 and Zoo3_Ind1_su and Zoo3_Ind3_wi (p: <0.01). The time series datasets were also tested for differences in Shannon index and showed strong significance (ANOVA: F=6.74, *p*<0.001). After testing for differences between the seasons and using only those individuals with both summer and winter samples, no significant differences were detected in richness (Kruskal–Wallis: chi-squared=1.02, p=0.31) or Shannon index (Kruskal–Wallis: chi-squared= 0.09, p=0.77).

Next, beta diversity was tested. Using PERMANOVA, significant differences between the time series datasets regarding their composition were found (*p*: <0.001). Using Multi-Response Permutation Procedure (MRPP), we found significant differences between the seasonal time series datasets for summer and winter (*p*: 0.001). All statistical values are shown in **Table 1**.

**Table 1:** Summary of multivariate test statistics assessing seasonal variation in microbial community composition.

Test	Variable	Statistic	Effect size	p-value
PERMDISP	Time-series	F=1.69	//	0.115
PERMANOVA	Time-series	F=2.58	R²=0.19	0.001 ***
PERMDISP	Season	F=20.481	//	0.001 ***
MRPP	Season	Observed delta=0.208 Expected delta=0.218	A=0.047	0.001 ***

*** indicates highly significant differences (p<0.001).

This analysis included only samples from individuals for which summer and winter samples were available. In conclusion, richness, Shannon index, and sample composition differed significantly between the time series datasets. The two assessed seasons showed no differences in diversity (richness and Shannon index) but did differ significantly in sample composition.

Richness (**Figure 1a**) shows high variation between the time series datasets. The highest variation was observed among the three individuals from Zoo3, whereas the pooled individuals from Zoo2 showed much less variation. The highest mean was detected in Zoo3_Ind1_summer. The Shannon index (**Figure 1b**) in Zoo3 shows the highest variation among samples from the same zoo. Zoo3 contained both the highest (Zoo3_Ind1_summer) and lowest (Zoo3_Ind2_winter) mean values. The beta diversity assessment revealed significant differences in the microbial composition of the time series datasets (*p*<0.001) and between the seasonally sorted summer and winter samples (*p*=0.001). To further understand the difference in beta diversity between the two seasons, microbial composition and abundance were assessed.

### 3.2 Time series composition

**Figure 2:**
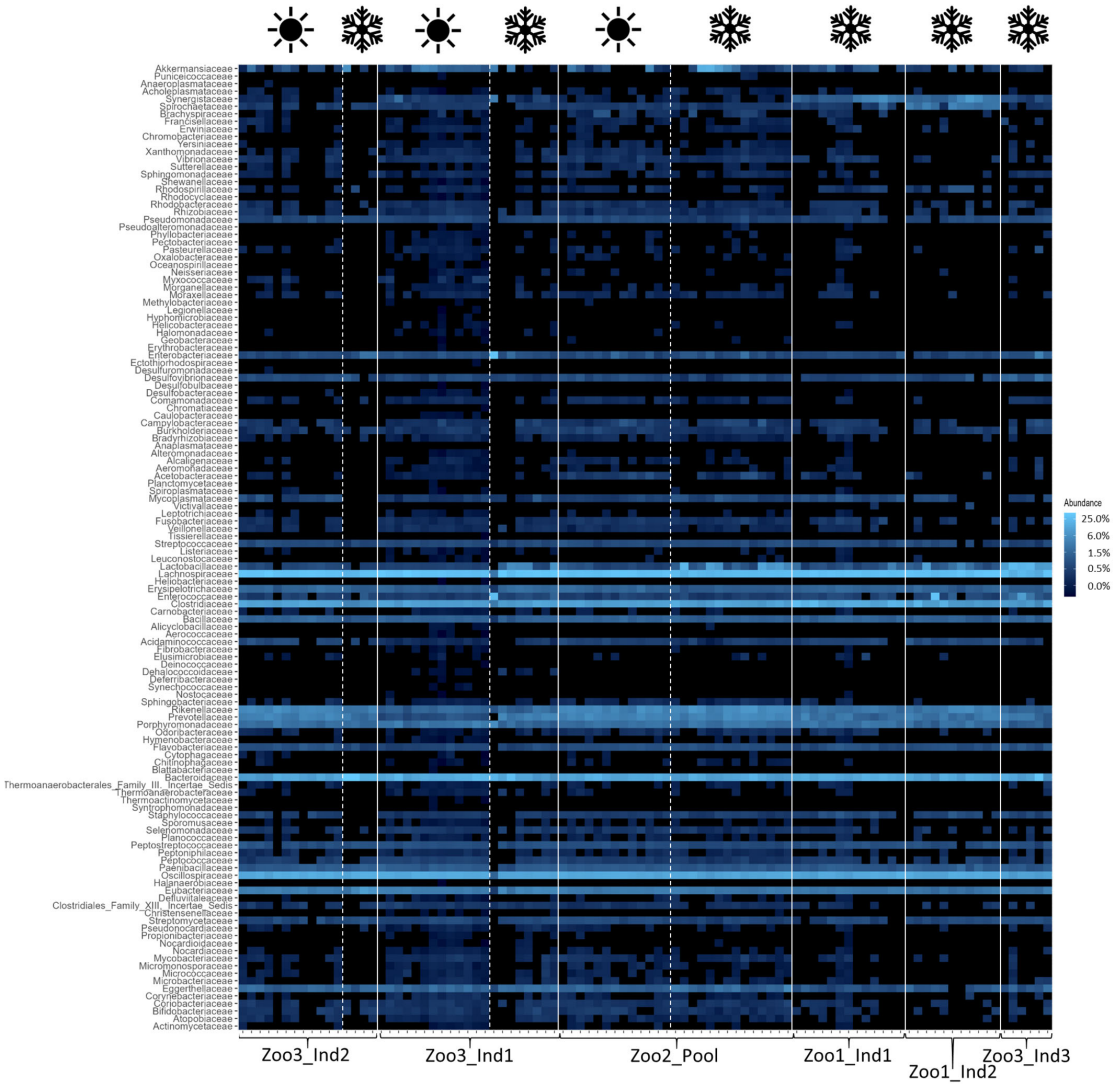
Heatmap of bacterial taxa across all samples. The white lines distinguish individuals and the white dashed lines distinguish seasons (as indicated with the symbols above; winter=snowflake and summer=sun). The relative abundances are depicted, with the color scheme specified in the legend on the right. The blue color increases in lightness with increasing abundance of the taxa.

The bacterial composition of each time series dataset was assessed and depicted in a heatmap (**Figure 2**) to display the most abundant taxa and their respective co-occurrence. The most prominent core bacteria—defined as taxa that occur in every sample—are shown with a connecting light blue line. Next, the average proportion of each core bacterial family out of all bacterial taxa is provided with standard deviation and coefficient of variation (cv), which are depicted in brackets after the standard deviation. The most abundant taxon was Lachnospiraceae, which constituted between 22.14 ± 5.97% (0.27) and 31.36 ± 02.73% (0.09). This was followed by Bacteroidaceae, which represented between 11.35 ± 2.83% (0.25) and 28.05 ± 10.29% (0.37), and Oscillospiraceae, which comprised between 14.58 ± 1.87% (0.13) and 10.42 ± 1.21% (0.12) of the sifakas’ microbiota. The proportion of Clostridiaceae ranged from 5.21 ± 1.11% (0.21) to 16.2 ± 4.95% (0.31). The following microbial families all occurred in less than 6% but nevertheless contributed to the core microbiota: Erysipelotrichaceae, which ranged between 1.00 ± 0.17% (0.17) and 1.59 ± 0.57% (0.36); Bacillaceae, which ranged between 0.79 ± 0.13% (0.16) and 1.57 ± 0.40% (0.25); and Rikenellaceae, which constituted between 1.43 ± 0.38% (0.27) and 5.56 ± 1.53% (0.27). Further, Porphyromonadaceae accounted for 1.32 ± 0.48% (0.36) up to 2.67 ± 0.79% (0.29), and lastly, the bacterial percentage of Eubacteriaceae in each sample ranged between 2.25 ± 0.45% (0.19) and 6.05 ± 2.91% (0.48).

**Figure 3:**
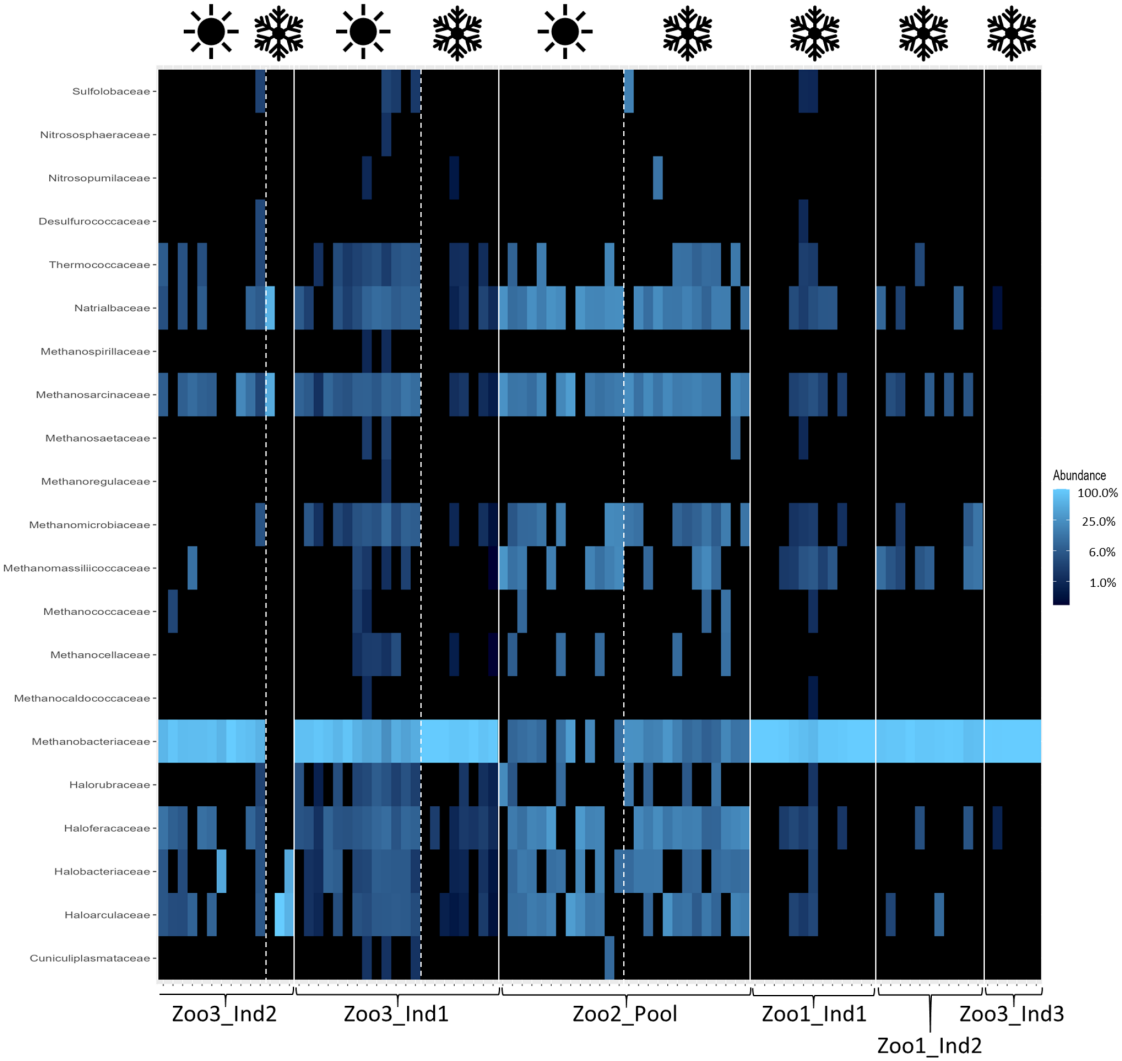
Heatmap of archaeal taxa across all samples. The white lines distinguish individuals and the white dashed lines distinguish seasons (as indicated with the symbols above; winter=snowflake and summer=sun). The relative abundances are depicted, with the color scheme specified in the legend on the right. The blue color increases in lightness with increasing abundance of the taxa.

**Figure 4:**
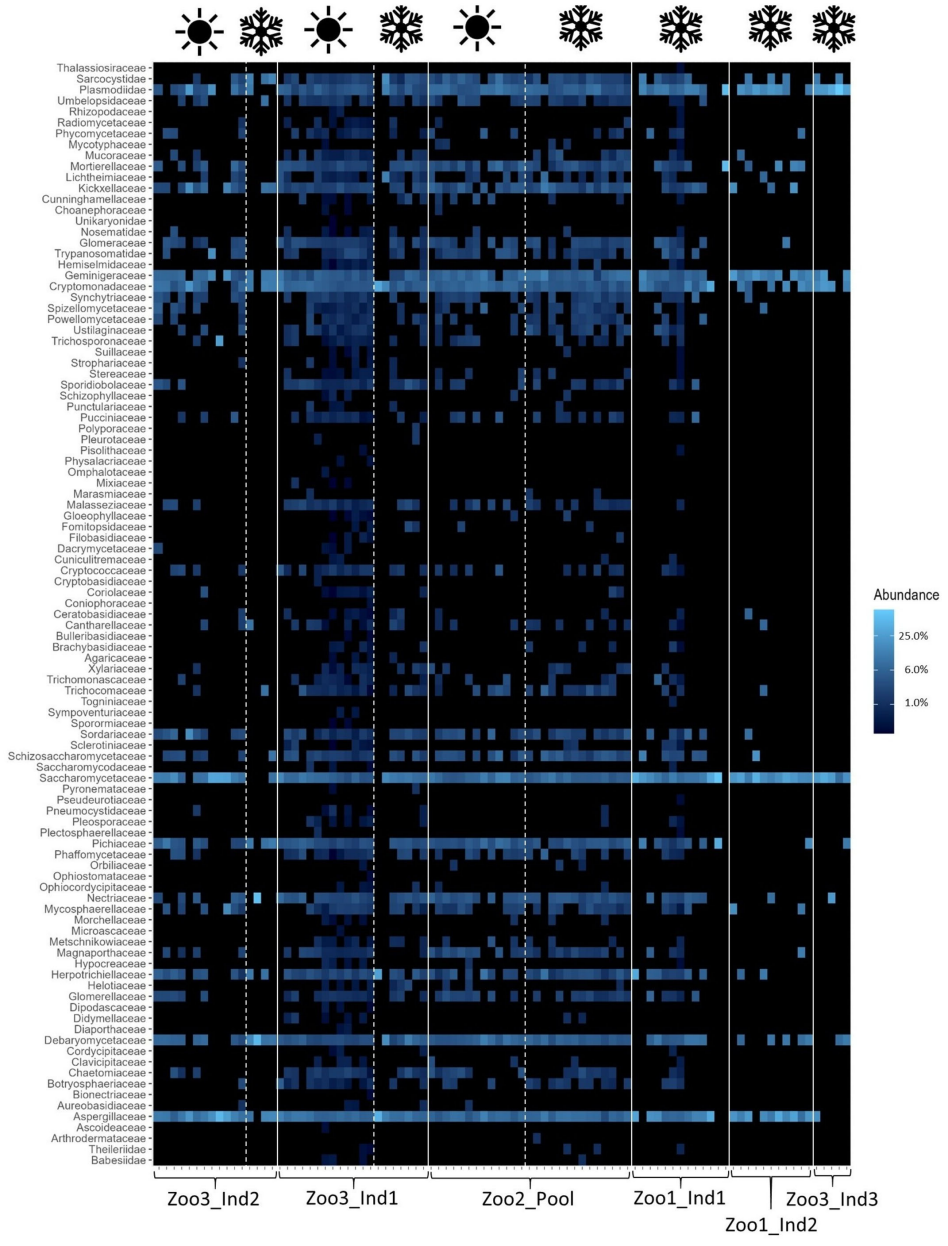
Heatmap of eukaryotic taxa across all samples. The white lines distinguish individuals and the white dashed lines distinguish seasons (as indicated with the symbols above; winter=snowflake and summer=sun). The relative abundances are depicted, with the color scheme specified in the legend on the right. The blue color increases in lightness with increasing abundance of the taxa.

Contrary to the more commonly applied method of 16S rRNA sequencing, this study included fungal and archaeal taxa in its analysis and detected the following families. **Figure 3** shows the archaeal families from each time series dataset. The most prominent and abundant taxon was the family Methanobacteriaceae. This family’s percentage out of all archaeal families detected ranged from 8.92 ± 9.37% (1.05) to 99.67 ± 0.73% (0.007). Regarding eukaryotic taxa, including fungal taxa, the detected families and their abundance are depicted in **Figure 4**. The four most prominent and abundant families, with their proportional range of all eukaryotic families, were as follows: Aspergillaceae constituted on average between 3.65 ± 7.31% (2.00) and 17.03 ± 8.93% (0.52). The family Debaryomycetaceae represented eukaryotic taxa with a proportional range from 3.96 ± 2.97% (0.75) to 3.77 ± 4.74% (1.26). Additionally, Geminigeraceae constituted between 3.17 ± 2.49% (0.79) and 12.75 ± 6.00% (0.47) of all eukaryotes. Lastly, Saccharomycetaceae comprised between 2.20 ± 3.41% (1.55) and 21.64 ± 8.52% (0.39) of the dataset.

## 4 Discussion

The aim of this study was to characterize the fecal microbiota of the critically endangered lemur species *Propithecus coquereli* and to test for individual and seasonal differences among the seven individuals. The natural diet of multiple members of the genus *Propithecus* consists mainly of leaves, some fruit, and marginal amounts of plant bark (National Research Council, 2003). The diet in human care settings assessed in this study was of similar composition. The zoos fed a mixture of foliage—fresh in the summer and supplemented with frozen leaves during the winter season—along with vegetables and seeds. Cultivated fruits have a much higher sugar content and differ greatly in nutrient composition compared to their wild counterparts (Milton, 1999). Therefore, fruit has been widely replaced by cultivated vegetables, which are higher in fiber and lower in sugar.

The results indicate that the season and the concurrent dietary changes do not create a decline in diversity, either in the number of taxa detected or in their abundance. However, the microbial profile of each season was significantly different. These findings are consistent with previous research. For example, a study on seasonal effects on primate microbiota indicated only minor impacts from seasonal temperature fluctuations but a stronger impact caused by changes in available diet in the wild. Vegetation varies between seasons in natural habitats, resulting in correlational microbiome adaptations. This change in diet can be mitigated in captivity by establishing stable food resources, minimizing seasonal effects (Baniel et al., 2021). Additionally, the high variability among samples in this study confirms previous findings, as the gut microbiome of *Propithecus coquereli* has been recognized as highly variable and diverse compared to other primate species. The high amounts of fiber consumed by this folivore generate a multitude of fermentation products and thus support high bacterial diversity (Greene et al., 2020; McKenney et al., 2015). This is further visualized in the heatmaps (**Figures 2**–**4**), which highlight differences in less abundant microbial families and show strong consistency in the main taxa across both seasons. The taxonomic identification and composition analysis revealed Lachnospiraceae, Bacteroidaceae, Oscillospiraceae, and Clostridiaceae as the most abundant bacterial taxa. Furthermore, Methanobacteriaceae was detected as the main archaeal family, and Aspergillaceae, Debaryomycetaceae, Geminigeraceae, and Saccharomycetaceae were identified as the main eukaryotic families in the time series datasets. The most abundant bacterial family in this study, Lachnospiraceae, is commonly found in primate gastrointestinal tracts, and members of this taxon are known to play a key role in the digestion of dietary fiber by converting it into SCFA such as butyrate and promoting healthy and functional intestinal epithelial cells (Vacca et al., 2020; Zaplana et al., 2023). A similar function is described for members of the Bacteroidaceae, which also participate in the degradation of carbohydrates into SCFA such as acetate and propionate and therefore assist the host in proper food digestion and nutrient processing. Moreover, Bacteroidaceae taxa have been shown to stimulate and support the host immune response to avoid pathogen colonization, potentially providing digestive and health-related benefits (Wexler, 2007; Zafar and Saier, 2021). Focusing on the large amount of cellulose that makes up the main part of the sifakas’ diet, Oscillospiraceae also plays a major role in digesting these components. This bacterial family is capable of degrading complex carbohydrates such as cellulose (La Reau and Suen, 2018). As a folivore species, *Propithecus coquereli* consumes large quantities of leaf matter containing high levels of cellulose but depends on endosymbiotic microbes such as species of the Oscillospiraceae family to digest and utilize this nutrient. Several low-abundance families found in this study are also linked to host metabolic health by providing the host with energy, assisting colonic motility, immunomodulation, and suppression of inflammation. These include Rikenellaceae, Porphyromonadaceae, and Erysipelotrichaceae (Lu et al., 2016; Tavella et al., 2021).

The sequencing method used in this study also detected archaea and eukaryotic fungal taxa in the fecal samples. The link between variation of micro eukaryotic communities in lemur gastrointestinal tracts remains unclear (Donohue et al., 2023). For example, Methanobacteriaceae comprise microbes that create methane in the gastrointestinal tract by reducing CO_2_ using hydrogen (Oren, 2014). By removing hydrogen from the gut, they regulate hydrogen accumulation, which can negatively impact the productivity of various gut microbiota, hence improving microbiome functionality (Guindo et al., 2020). This family further stood out, as members of this family have been linked to multiple metabolic diseases, such as adiposity and anorexia and their occurrence has been assumed to influence host health (Chaudhary et al., 2018). As an archaeal family, the abundance of this taxon is considered to be influenced directly by host diet (Thomas et al., 2022). The two sampling sets with the highest Methanobacteriaceae proportion are Zoo3_Ind1_winter and Zoo3_Ind3_winter, which were both fed a similar diet, with the absence of nuts and leaf eater pellets, which were fed to those individuals during the summer sampling period. A main fungal taxon Aspergillaceae, has been described as a main family in other herbivorous primates (Li et al., 2018). However, the fungal taxon Saccharomycetaceae, found in two of the time series datasets, is suggested to provide the host with beneficial impacts on host immune response (Ortuño et al., 2002), whereas the complex interaction between host and fungi in the gut microbiome is still under investigation (Wheeler et al., 2017). The sample size of seven individuals in this study was quite low, making statistical evaluation challenging. Nevertheless, the authors considered the efforts worthwhile to increase knowledge and understanding of this critically endangered primate species and hope to contribute to captive conservation efforts. In summary, the gut microbiota of *Propithecus coquereli* appears well adapted to a folivorous diet, with key microbial families supporting the efficient degradation of plant material and the continuous production of short-chain fatty acids (SCFAs), which are essential for maintaining colonic health (Wong et al., 2006). Regarding composition and microbial diversity, *Propithecus coquereli* displayed high levels of variability and diversity, as expected from a herbivorous mammal species (Zoelzer et al., 2021). The season did not create a decline in diversity, indicating that the supplementation of frozen foliage supplementation during the winter months had no significant effect on alpha diversity of the fecal microbiota of *Propithecus coquereli*. Additionally, we observed very similar average body weights in all individuals sampled during the summer (N=4, mean bodyweight= 4.34 ± 0.38 kg) and those sampled during winter (N=7, mean bodyweight= 4.29 ± 0.35 kg). The reduced number of study objects was caused by the death of one individual and the fact that one zoo could only be sampled during winter. This consistent body weight further indicates homogenous physiology throughout the year. Therefore, maintaining a healthy gut microbiome is likely to impact overall host health and well-being positively and should be considered in animal husbandry. The community of these main families shapes an effective gut microbiota for a folivorous species such as *Propithecus coquereli* by enabling efficient digestion of plant derived food items, particularly adapted to fiber degradation (Greene et al., 2020). The analysis of the fecal microbiota in populations of *Propithecus coquereli* and the assessment of the possible impact of dietary variation aim to provide useful information for captive breeding and conservation efforts by the zoo community. These results suggest that local foliage may be fed year-round, providing fiber-rich diets to the folivore without causing microbial diversity decline. Understanding the common composition of microbes may also be useful for fecal-sample-based health monitoring and early discovery of dysbiosis or gut health issues.

## 5 Conclusions

To the best of our knowledge, this was the first study to assess the fecal microbiota of the seven individuals of *Propithecus coquereli* housed in EAZA zoos and to test for the impact of seasonal dietary changes. We showed a highly variable fecal microbiota with no distinct microbial profiles per individual. With time series of up to 14 days, we found no oscillation or repeating patterns, which highlights the importance of longitudinal studies when it comes to fecal microbiota assessments of species with such variable microbial composition. We found no significant differences in alpha diversity between the summer and winter samples. Therefore, we found no indication that supplementation with frozen foliage during the winter months influenced a decline in the gut microbiota that could potentially allow for pathogen colonization and disease. For follow-up studies, it would be interesting to increase the length of each time series and to provide more consistent sampling in both seasons.

## Data Availability

The names of the repository/repositories and accession number(s) can be found below: https://www.ncbi.nlm.nih.gov/bioproject/PRJNA1301845/.
